# Recent Advances in 1,8-Naphthalimide-Based Supramolecular Gels (2020–2026)

**DOI:** 10.3390/gels12100933

**Published:** 2026-10-16

**Authors:** Abelardo Sánchez Oliva, Iván Grande Morales, Eva Luna Golvano, Iván Torres-Moya

**Affiliations:** 1 Department of Inorganic, Organic Chemistry and Biochemistry, Faculty of Chemical Science and Technologies, University of Castilla-La Mancha-IRICA, 13071 Ciudad Real, Spain; abelardosanchez98@gmail.com (A.S.O.); ivan.grande@uclm.es (I.G.M.); eva.luna@alu.uclm.es (E.L.G.); 2 Department of Inorganic Chemistry, Organic Chemistry and Biochemistry, Faculty of Pharmacy, University of Castilla-La Mancha, Av. Dr. José María Sánchez Ibáñez, s/n, 02071 Albacete, Spain

**Keywords:** supramolecular gels, naphthalimides (NI), self-assembly, aggregation-induced emission (AIE), stimuli-responsive materials, bioimaging

## Abstract

Naphthalimide (NI) derivatives constitute attractive building blocks for the construction of fluorescent supramolecular gels owing to their structural versatility, remarkable photophysical properties, and ability to participate in non-covalent interactions. These properties prompted us to write this review, that provides a focused overview of the representative advances reported between 2020 and 2026 in NI-based supramolecular gels, covering the evolution from low-molecular-weight organogelators and metallogels to peptide-based and water-compatible hydrogels. Particular attention is paid to the influence of molecular design on self-assembly and gelation, including the contribution of hydrogen bonding, π–π stacking, metal coordination, and other intermolecular interactions to the formation of fibrillar supramolecular networks. The close relationship between aggregation and the photophysical response of the NI chromophore is also discussed, highlighting phenomena such as aggregation-induced and aggregation-enhanced emission and gelation-induced fluorescence enhancement. These properties have enabled the development of stimuli-responsive materials displaying fluorescence modulation and gel–sol or gel–gel transitions, particularly in response to metal ions and chemical stimuli. Recent studies also further demonstrate a transition toward more complex functional systems, including fluorescent peptide hydrogels and thixotropic materials with potential biomedical applications. The main applications developed during this period, ranging from chemical sensing to smart fluorescent materials, bioimaging, and biomedical systems, are critically analyzed. Finally, current limitations and future opportunities for NI-based gels are discussed, with particular emphasis on the development of red and near-infrared emissive systems and advanced soft photonic materials.

## 1. Introduction

Gels constitute a fascinating class of soft materials that combine the structural characteristics of solids with the dynamic properties of liquids. They consist of a three-dimensional network capable of immobilizing a large amount of solvent while maintaining a solid-like macroscopic appearance [1,2,3]. This unique combination of properties has stimulated considerable interest in gels for applications spanning materials science, sensing, drug delivery, tissue engineering, and other biomedical technologies [4,5,6,7,8,9,10,11,12,13,14,15,16,17,18,19,20]. Depending on the nature of the liquid phase, these materials can be broadly classified as hydrogels or organogels when water or an organic solvent, respectively, constitutes the main liquid component. Beyond their conventional classification, the chemical nature of the network-forming species and the interactions responsible for network formation are particularly relevant, as they ultimately determine the mechanical, morphological, responsive, and functional properties of the resulting gel.

Among the different families of gels, supramolecular gels have attracted increasing attention because their three-dimensional networks are generated through the self-assembly of molecular building blocks rather than permanent covalent crosslinking. In particular, low-molecular-weight gelators (LMWGs) can hierarchically self-assemble through a combination of non-covalent interactions, including hydrogen bonding, π–π stacking, van der Waals forces, solvophobic interactions, and electrostatic interactions, as well as through metal–ligand coordination interactions [21,22].

The possibility of encoding functionality at the molecular level is one of the most appealing features of LMWGs. Rational incorporation of chromophores, recognition units, coordinating groups, or biologically active moieties into the gelator structure enables the resulting supramolecular network to perform functions beyond simple solvent immobilization. However, the incorporation of additional functional units may also alter molecular packing and interfere with the intermolecular interactions responsible for self-assembly, potentially compromising or even suppressing gelation. Therefore, achieving an appropriate balance between functionalization and gel-forming ability remains an important challenge in the design of multifunctional supramolecular gelators. Fluorescent LMWGs are particularly attractive because molecular self-assembly can directly influence their electronic and excited-state properties. The proximity and specific arrangement of chromophores within supramolecular aggregates may result in fluorescence quenching, spectral shifts, energy or electron transfer processes, or enhanced emission upon aggregation. Thus, the photophysical response itself can provide valuable information about the self-assembly process while simultaneously introducing optical functionality into the material.

Within the wide variety of fluorophores employed for the construction of functional molecular materials, 1,8-naphthalimide (NI) derivatives have emerged as particularly versatile scaffolds. Their widespread use arises from a combination of favorable characteristics, including high chemical and photochemical stability, generally strong fluorescence, relatively high fluorescence quantum yields, and straightforward structural modification [23,24,25,26,27,28,29,30]. The NI framework is an electron-deficient moiety, and through an appropriate functionalization of the aromatic core with electron-donating substituents it can generate donor–acceptor architectures characterized by intramolecular charge-transfer (ICT). Consequently, their absorption and emission properties can be finely modulated through molecular design as well as by changes in the surrounding environment. Modification of the imide nitrogen additionally provides a convenient strategy for tuning solubility, introducing recognition or targeting units, and controlling intermolecular interactions without necessarily disrupting the principal electronic characteristics of the chromophore. These features have established NIs as important fluorophores in fields ranging from chemical sensing and molecular probes to bioimaging and advanced functional materials in organic photonics, electronics or photovoltaics [23,24,25,26,27,28,29,30].

Importantly, the same structural characteristics that determine the photophysical properties of NIs also make them attractive components of supramolecular gelators. Their extended aromatic surface can promote π–π interactions, while suitable functionalization with amide, amino acid, peptide, hydroxyl, carboxylate, or coordinating groups introduces additional hydrogen-bonding, electrostatic, or metal-coordination interactions capable of directing hierarchical self-assembly. The resulting balance between aromatic interactions, peripheral functional groups, solvent affinity, and molecular geometry determines whether the molecules remain dissolved, precipitate, crystallize, or organize into extended fibrillar networks capable of gel formation. Moreover, incorporation of the NI chromophore provides an optical reporter that is highly sensitive to changes in its local environment and aggregation state. This has enabled the development of NI-based gels exhibiting aggregation-induced emission (AIE), aggregation-enhanced emission (AEE), gelation-induced fluorescence enhancement, fluorescence quenching, and reversible optical responses associated with gel–sol or gel–gel transformations. The combination of self-assembly and photophysical responsiveness is therefore particularly advantageous for constructing multifunctional soft materials in which molecular recognition can be translated into readily observable optical and macroscopic responses.

Over the last few years, the scope of NI-based supramolecular gels has progressively expanded. Initial studies predominantly focused on fluorescent LMW organogelators and metallogels, in which molecular self-assembly and gelation could be modulated by coordination, redox processes, pH, or the presence of specific metal ions. These systems demonstrated the potential of NI-containing gels for fluorescence sensing, ion recognition, and responsive surfaces. More recently, molecular design has increasingly incorporated amino acids and short peptide sequences, facilitating self-assembly in aqueous environments and leading to fluorescent peptide-based hydrogels with improved biocompatibility, aggregation-enhanced emission, and interesting rheological properties. In particular, the emergence of thixotropic and potentially injectable NI-containing hydrogels represents an important step toward their exploitation in bioimaging and other biomedical applications. This evolution illustrates how the NI unit has progressed from acting primarily as a fluorescent reporter within an organogelator to becoming an integral component of increasingly sophisticated multifunctional supramolecular materials.

**Figure 1:**
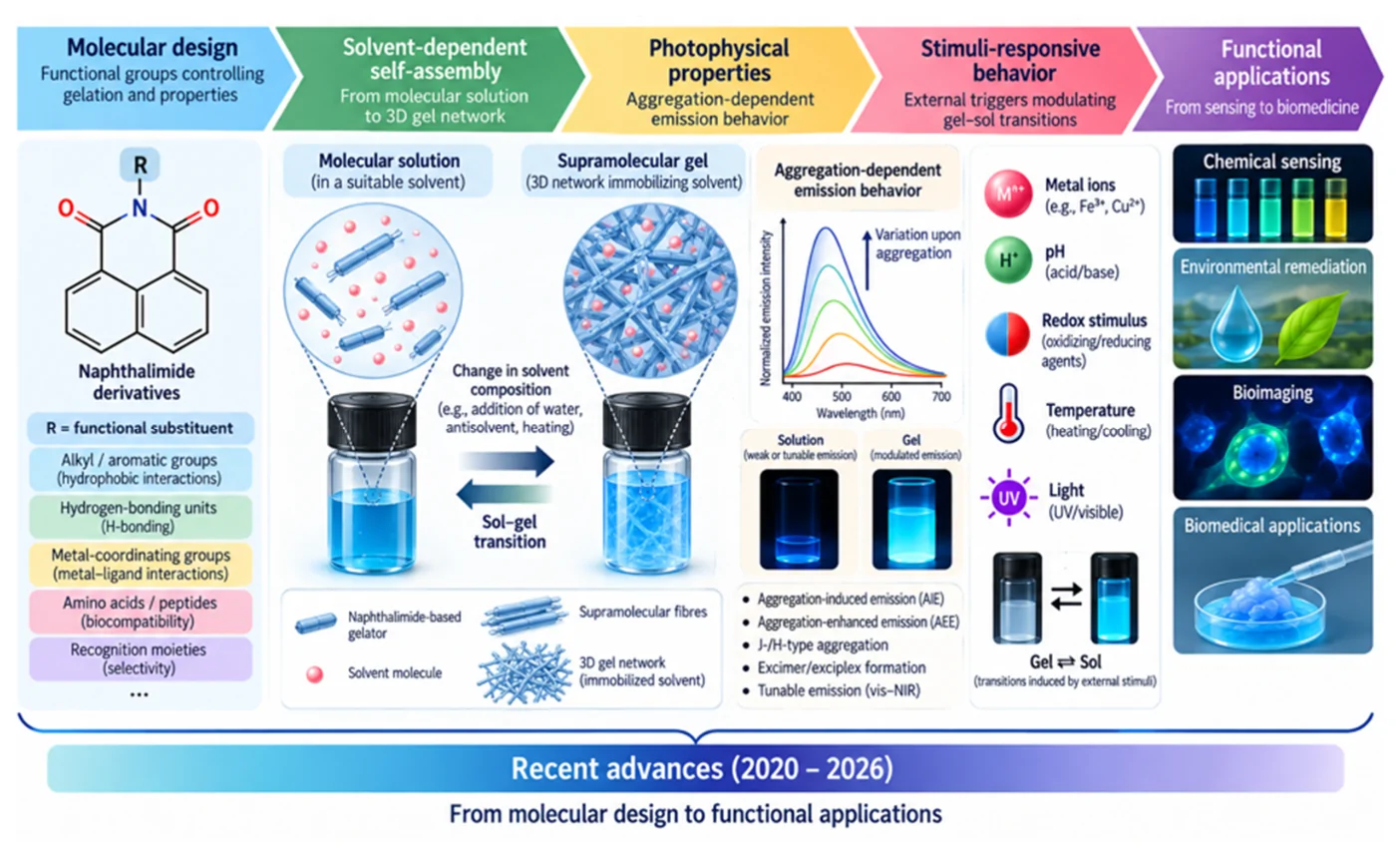
Schematic goal of this review shows the importance of NI in the design of advanced functional gels.

Despite the substantial research devoted to both supramolecular gels and 1,8-naphthalimide fluorophores independently in previous years [31,32,33,34,35,36], recent developments specifically concerning NI-based supramolecular gels remain comparatively scattered. The present review therefore provides a focused analysis of representative advances reported between 2020 and 2026, with particular emphasis on the relationship between molecular structure, intermolecular interactions, self-assembly, gel formation, and photophysical behavior. The selection of representative studies was based on their relevance to the molecular design of NI-based gelators, the diversity of supramolecular self-assembly strategies, their photophysical properties, and their potential functional applications. Particular attention was given to studies illustrating distinct gelation mechanisms and structure–property relationships across different classes of NI-based supramolecular gels. Recent NI-containing organogels, metallogels, and hydrogels are discussed from a structure–property perspective, highlighting aggregation-dependent emission phenomena and stimuli-responsive behavior. Their emerging applications in chemical sensing, smart fluorescent materials, bioimaging, and biomedical systems are subsequently examined (Figure 1). Finally, the current limitations of these materials and future opportunities for the development of red- and near-infrared-emissive gels, more quantitatively understood excited-state processes, and advanced NI-based soft photonic materials will also be critically considered at the end of this article.

## 2. Most Representative Examples of the Employment of NI Gels in Recent Years

In this review, a representative set of 12 research articles from 2020 to 2026 in which NIs have been employed as supramolecular gels have been selected. First of all, the oldest example for this review illustrating the potential of NI as a multifunctional building block for supramolecular hydrogels was reported by Rizzo et al. in 2020 [37]. The authors designed a family of fluorescent imidazolium salts, **[CnNIim][X]**, in which a chloro-substituted NI fluorophore was connected to an imidazolium unit through a propyl spacer, while the alkyl chain length (C8–C14) and counteranion (Br^−^ or gluconate) were systematically varied. In this molecular architecture, the NI moiety played a dual structural and photophysical role: its aromatic surface contributed to the intermolecular interactions involved in self-assembly, whereas its aggregation-dependent fluorescence provided an optical reporter of the organization process. Gelation was strongly governed by the hydrophilic–hydrophobic balance of the entire molecule. Thus, **[C8NIim][Br]** was unable to gel, whereas increasing the alkyl chain length markedly improved gelation, with **[C14NIim][Br]** forming hydrogels in several aqueous media. This behavior was attributed to the cooperative contribution of van der Waals interactions from the long alkyl chains together with hydrogen bonding and π–π interactions involving the aromatic units. The nature of the counteranion also influenced self-assembly, since replacement of bromide by the more strongly hydrated gluconate anion reduced the gelation ability and increased the critical gelation concentration. These observations already highlighted an important design principle for NI-based gelators: although the aromatic NI core provides a suitable platform for supramolecular organization, efficient gelation requires a finely balanced contribution from the peripheral hydrophobic and ionic functionalities [37].

**Figure 2:**
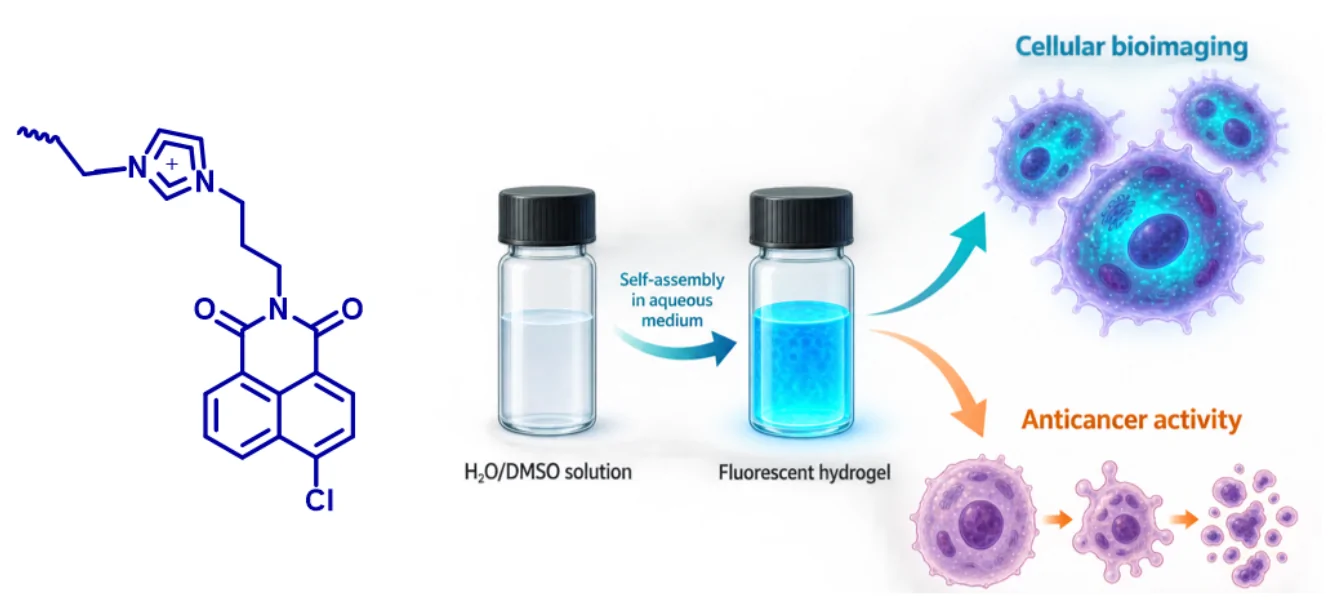
Original schematic representation of the NI–imidazolium system, illustrating the formation of a fluorescent supramolecular hydrogel in an H_2_O/DMSO medium and its reported applications in cellular bioimaging and anticancer activity. Recreated from Ref. [37] with ChatGPT Sol 6.1.

The NI core was particularly relevant to the optical functionality of these hydrogels. Increasing the water fraction in DMSO/H_2_O mixtures promoted aggregation of the NI chromophores, accompanied by a bathochromic shift in absorption and a pronounced enhancement of fluorescence, which the authors interpreted as being consistent with J-type aggregation and aggregation-induced emission (AIE) behavior. Importantly, significant blue fluorescence was retained after gel formation, allowing the supramolecular material to combine gelation with optical functionality (Figure 2). Beyond its contribution to self-assembly and fluorescence, the NI–imidazolium platform also endowed the systems with biological activity. The salts were efficiently internalized by HeLa, HCT116, and MCF7 cancer cells and could be visualized through their intrinsic fluorescence, while micromolar cytotoxicity was observed toward the investigated cancer cell lines (Figure 2). Particularly relevant was the gluconate-containing derivative, which displayed improved selectivity toward cancer cells. Overall, this study demonstrated at an early stage that the NI scaffold could serve simultaneously as an aggregation-sensitive fluorophore, a contributor to supramolecular organization, and a functional component of theranostic soft materials, establishing a connection between molecular self-assembly, fluorescence and biological activity that would subsequently become increasingly relevant in NI-based hydrogel design [37].

**Figure 3:**
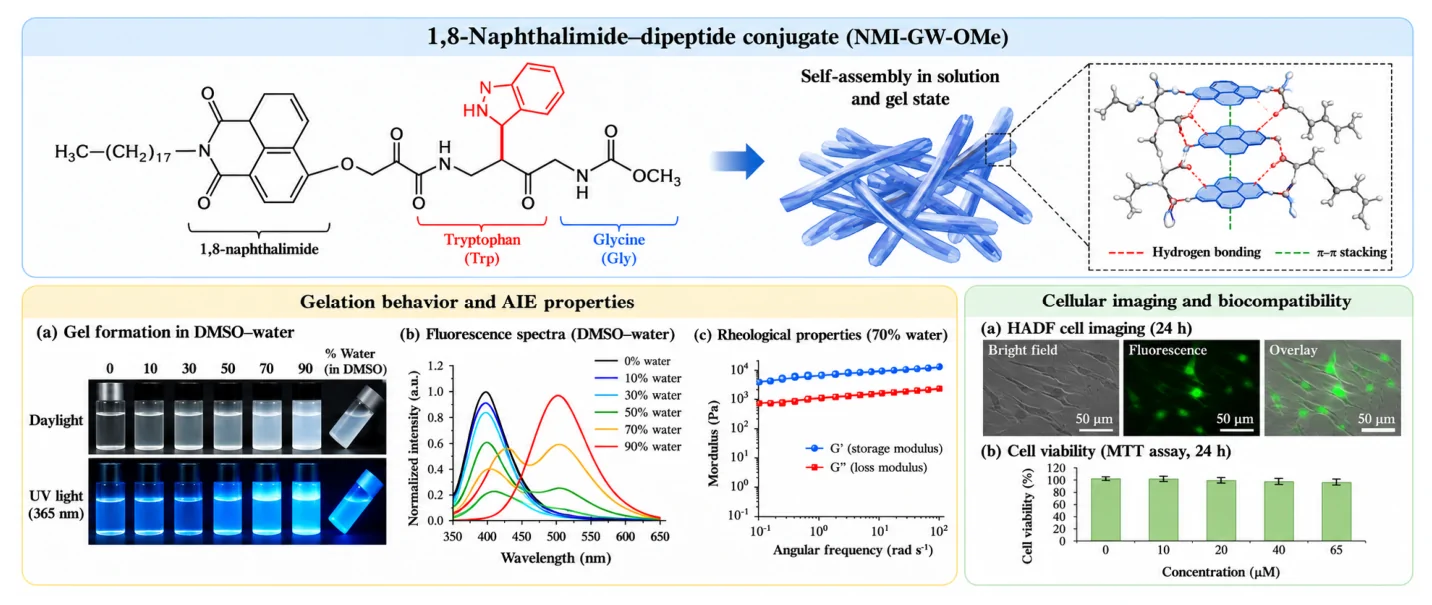
Schematic representation of the molecular structure and supramolecular self-assembly of the 1,8-naphthalimide–dipeptide conjugate (**NMI-GW-OMe**), highlighting the role of hydrogen bonding and π–π stacking interactions in the formation of nanofibrillar networks. Gelation behavior in DMSO/water mixtures, aggregation-induced emission (AIE) properties, and rheological characterization of the resulting gels are also illustrated. The potential application in bioimaging is demonstrated through fluorescence microscopy of human adult dermal fibroblast (HADF) cells, together with cell viability measurements. Recreated from Ref. [38] with ChatGPT Sol 6.1.

Also in 2020, Misra et al. reported the synthesis and supramolecular self-assembly of an NI–dipeptide conjugate (**NMI-GW-OMe**), incorporating glycine and tryptophan residues, which exhibited remarkable gelation and AIE properties (Figure 3). Single-crystal X-ray diffraction revealed a slipped molecular arrangement stabilized by intermolecular amide hydrogen bonding and aromatic π–π interactions, consistent with a J-type packing geometry in the crystalline state, while additional C–H···O interactions contributed to the observed supramolecular organization. Interestingly, the compound formed supramolecular gels in aromatic solvents and long-chain aliphatic alcohols upon sonication, with critical gelation concentrations ranging from 10 to 18 mM. Moreover, the addition of water to solutions of **NMI-GW-OMe** in water-miscible organic solvents, including DMSO, DMF, THF, and acetonitrile, induced gel formation through an antisolvent-driven self-assembly process. Rheological measurements confirmed the formation of self-supporting gels, with storage moduli (G′) exceeding loss moduli (G″) and G′ values on the order of 10^3^ Pa [38].

The photophysical properties were strongly influenced by the aggregation state. In DMSO solution, **NMI-GW-OMe** exhibited an emission maximum at 386 nm, which progressively red-shifted upon increasing the water content. At 60% water, a significant enhancement of fluorescence intensity was observed, accompanied by an emission band at 416 nm and a broad excimer-related contribution centered at 527 nm, characteristic of aggregation-induced emission behavior. Furthermore, time-resolved fluorescence measurements revealed an increase in the average excited-state lifetime from 2.3 ps in DMSO to 463 ps in DMSO/H_2_O (10:90, *v*/*v*). Transmission electron microscopy revealed nanofibrillar networks with average fiber diameters of 15.6 ± 3.1 nm and 19.0 ± 3.2 nm in DMSO/H_2_O and xylene, respectively. The potential biological applicability of this system was also demonstrated through fluorescence imaging of human adult dermal fibroblast (HADF) cells, which exhibited intracellular green fluorescence after incubation with the conjugate. Cell viability remained approximately 98% at 65 μM after 24 h, supporting its potential as a fluorescent probe for cellular imaging. Overall, this study highlights how peptide functionalization and hydrogen-bond-directed molecular packing can modulate the aggregation behavior of otherwise aggregation-quenched naphthalimide chromophores, enabling the development of luminescent supramolecular gels with potential bioimaging applications (Figure 3) [38].

Moving from fluorescent hydrogels toward metal-mediated supramolecular systems, Lovitt et al. reported in 2021 two 4-morpholino-NI derivatives (**L1** and **L2**) designed to combine the characteristic photophysical properties of the NI fluorophore with pH responsiveness and coordination-driven self-assembly. In **L1**, a 3-picolyl group was attached to the imide nitrogen, whereas this coordinating unit was replaced by a benzyl group in **L2**, providing a particularly informative structure–function comparison. The morpholino substituent at the 4-position acts as an electron-donating group and gives rise to the characteristic push–pull electronic structure and ICT emission of amino-substituted NIs Importantly, crystallographic analysis provided direct evidence for the involvement of the NI scaffold in supramolecular organization. The solid-state structure of **L1** displayed face-to-face π–π stacking between adjacent NI units, whereas crystals obtained following disruption of the metallogel exhibited offset head-to-tail π–π interactions between NI cores, with a centroid–centroid separation of 3.58 Å. These observations support an active structural role of the aromatic NI platform in addition to its function as a fluorescent reporter [39].

**Figure 4:**
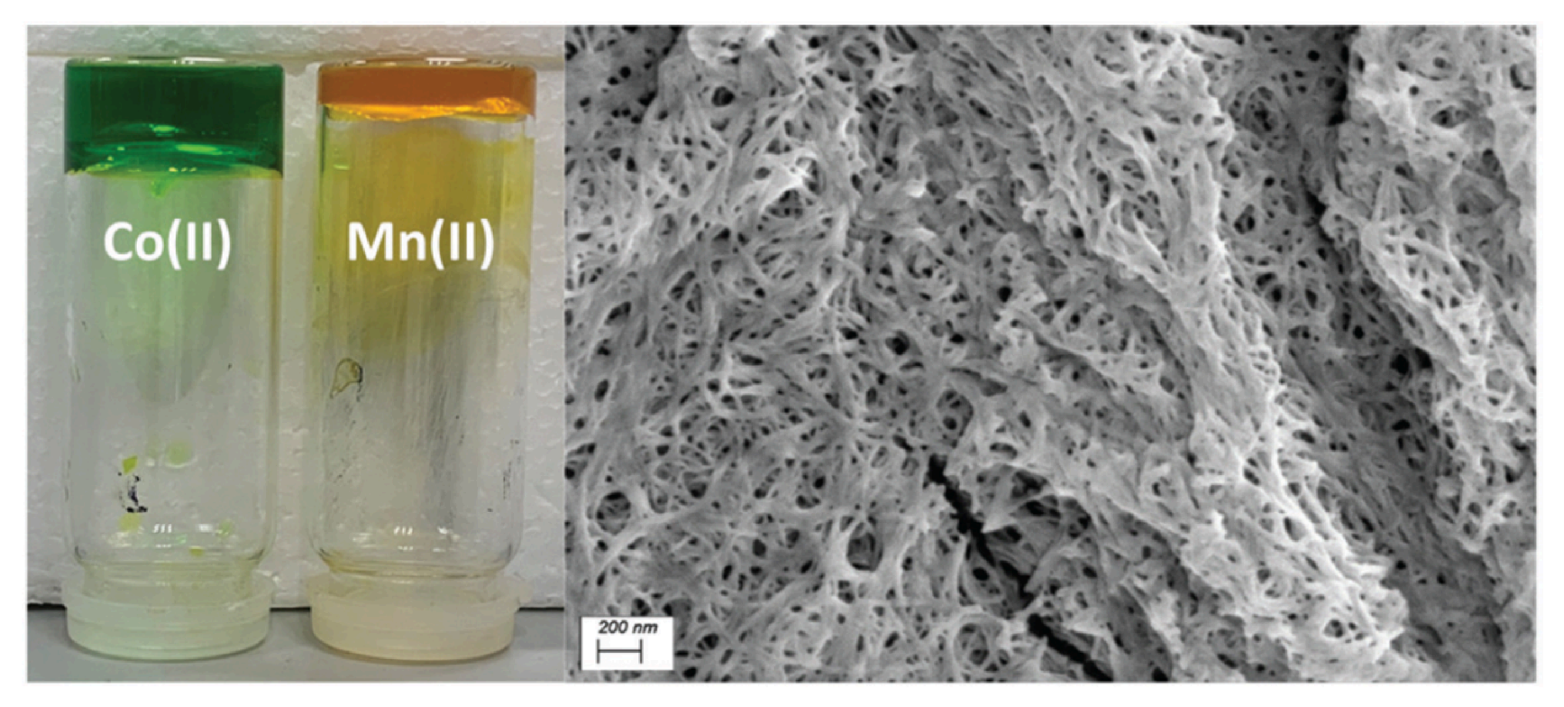
(**left**) **Co-L1** (gel 2) and **Mn-L1** (gel 1); (**right**) SEM image showing the fibrous morphology of the xerogel 2. Reproduced from Figure 11 of Ref. [39] under the terms of the Creative Commons Attribution 3.0 Unported license (CC BY 3.0).

Nevertheless, the comparison between **L1** and **L2** clearly demonstrated that NI stacking alone was insufficient to induce gelation. The treatment of the 3-picolyl-containing **L1** with Mn(II) or Co(II) chlorides in acetonitrile afforded stable metallogels, whereas the corresponding benzyl derivative **L2** produced amorphous or microcrystalline precipitates under analogous conditions, establishing metal coordination through the peripheral pyridyl functionality as a key requirement for metallogel formation. The resulting **Mn-L1** and **Co-L1** materials exhibited densely packed fibrous morphologies and reversible thermal responses, with gel–sol transition temperatures of 74–76 and 52–54 °C, respectively (Figure 4). Their dynamic supramolecular nature was further reflected in their response to acidic/basic stimuli and metal sequestration, as well as in rheological recovery experiments [39].

From a photophysical perspective, this study provides an interesting contrast with the AIE-active NI hydrogelators described in 2020. **L1** and **L2** did not exhibit conventional AIE upon increasing the water fraction in CH_3_CN/H_2_O mixtures; instead, aggregation resulted in decreased emission. Nevertheless, their fluorescence remained strongly coupled to supramolecular organization. In particular, **L1** underwent pH- and counterion-dependent aggregation accompanied by pronounced changes in absorption and fluorescence and the formation of micrometre-scale particles. Thus, the optical response could not be explained solely in terms of protonation/deprotonation of the molecular receptor but also involved changes in the aggregation state. Overall, this work illustrates a different strategy for exploiting NI in functional gels: rather than relying on aggregation-induced fluorescence enhancement, the NI core serves simultaneously as an ICT fluorophore and π-stacking supramolecular synthon, while an independently addressable coordinating group controls metal-induced gelation. This modular separation between the emissive, self-assembling and coordination functions provides an attractive design principle for stimuli-responsive luminescent metallogels.

**Figure 5:**
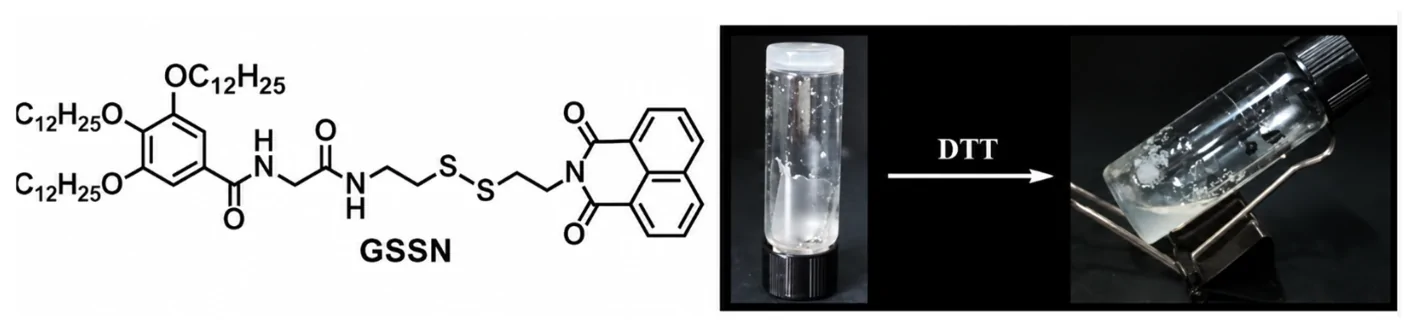
Molecular structure of the disulfide-containing NI-based gelator **GSSN** and schematic representation of its reduction-responsive gel-to-sol transition upon addition of dithiothreitol (DTT) [40]. Adapted with permission from Figure 1 and Figure 3 of Ref. [40]. Copyright © 2022 Wiley-VCH GmbH.

A different strategy toward stimuli-responsive NI-based gels was reported by Pei et al. in 2022 [40] through the design of gallic-acid-modified NI derivatives as LMWGs. Two structurally related compounds, **GSSN** and **GN**, were investigated, with **GSSN** incorporating a redox-responsive disulfide linkage (Figure 5). Both derivatives exhibited efficient gelation in selected organic solvents at relatively low concentrations, generating three-dimensional supramolecular networks. Spectroscopic and structural studies indicated that self-assembly was cooperatively driven by hydrogen bonding, π–π stacking between the aromatic moieties, and van der Waals interactions. In this molecular design, the NI core therefore contributed not only to the optical properties of the materials but also provided an extended aromatic surface that favored intermolecular association and organization of the gelator molecules. Particularly interesting was the behavior of **GSSN**, whose disulfide linkage endowed the resulting organogel with reduction responsiveness, allowing chemical modulation of the assembled state. In addition, the xerogel surfaces displayed pronounced hydrophobicity, suggesting their potential use as functional hydrophobic coatings. This study therefore broadened the scope of NI-based supramolecular gels beyond conventional fluorescent or ion-responsive systems, demonstrating how the NI scaffold can be integrated with a cleavable redox-active motif to generate stimuli-responsive soft materials with externally controllable self-assembly [40].

**Figure 6:**
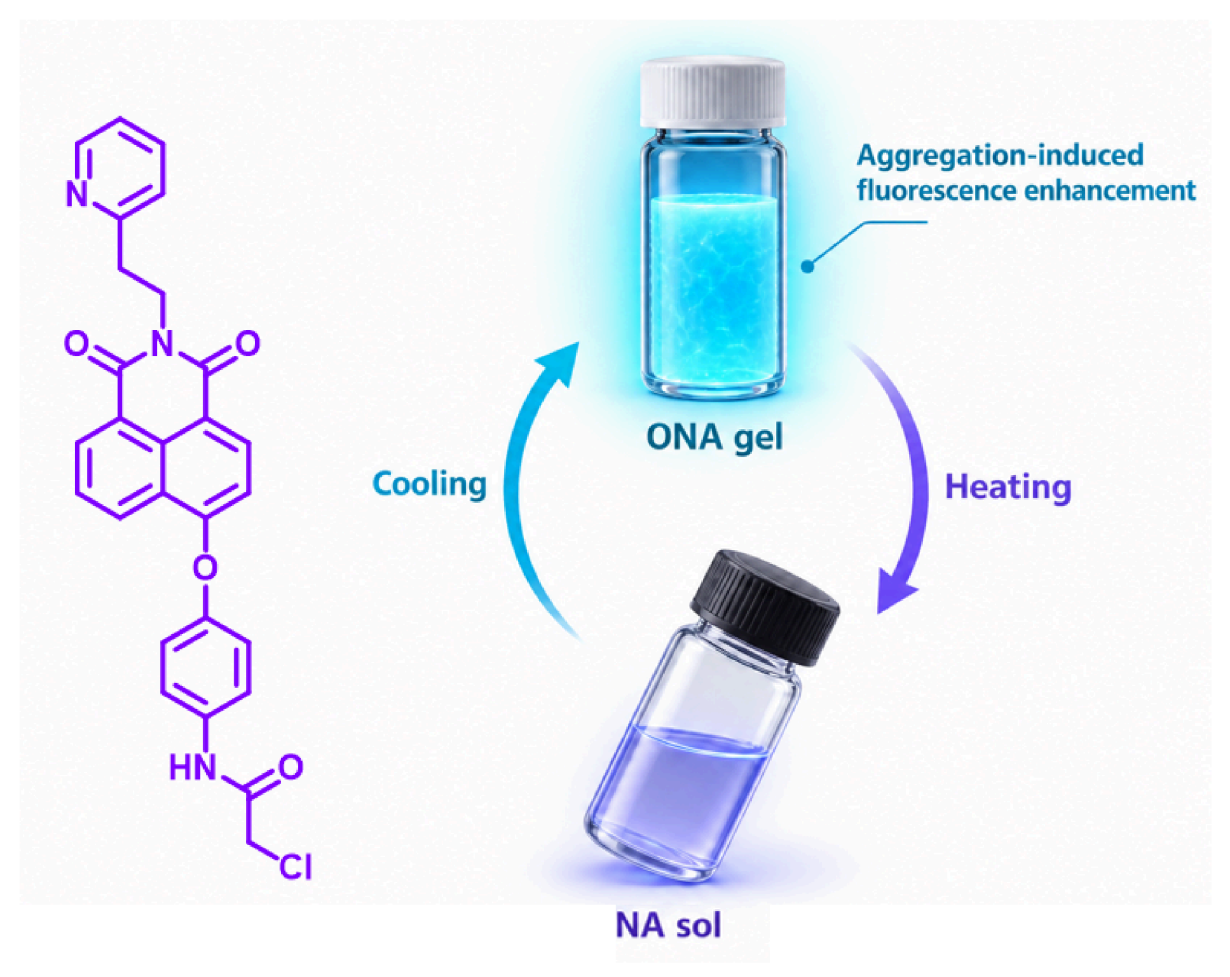
Original schematic representation of the molecular structure of gelator **NA** and its thermally reversible sol–gel transition, accompanied by aggregation-induced fluorescence enhancement. Recreated from Ref. [41] with ChatGPT Sol 6.1.

A further step toward stimuli-responsive NI-based gels was reported by Jiang et al. in 2022 [41] through the development of the LMWG **NA**, which combines an NI fluorophore with pyridyl and amide functionalities. In contrast to the coordination-driven metallogelation described by Lovitt et al., the metal-free NA molecule was itself capable of self-assembling in 1,2-propanediol to produce the supramolecular organogel **ONA**, with a critical gelation concentration of 1% (*w*/*v*) and a T_gel_ of 54 °C. The molecular design assigned complementary functions to the different structural components: the amide group promoted intermolecular hydrogen bonding, whereas the planar NI core provided both the π-surface required for aromatic interactions and the aggregation-sensitive fluorescent response (Figure 6). The involvement of hydrogen bonding was supported by variable-temperature ^1^H-NMR experiments, while XRD measurements provided evidence for π–π interactions within the xerogel. Thus, this system further illustrates the cooperative nature of NI-based gelation, in which the NI scaffold contributes to supramolecular organization but operates together with peripheral hydrogen-bonding units to stabilize the three-dimensional network [41].

Particularly noteworthy was the direct coupling between gelation and the photophysical behavior of the NI chromophore. Whereas the hot sol displayed relatively weak and broad emission, cooling below T_gel_ resulted in gel formation together with a pronounced fluorescence enhancement and a shift of the emission to 467 nm, which was assigned to aggregation-induced emission enhancement (AIEE). Solvent-dependent experiments and theoretical calculations indicated that **NA** undergoes a twisted intramolecular charge-transfer (TICT) process in solution, whereas aggregation restricts intramolecular motion and modifies the excited-state behavior. The authors further proposed that specific intermolecular π–π interactions between the planar NI cores account for the markedly blue-shifted emission observed in the condensed gel state. Therefore, unlike the non-AIE system reported in 2021, the NI unit in **ONA** acts simultaneously as a supramolecular π-stacking element and an aggregation-responsive fluorophore, directly translating molecular organization into a readily observable optical signal.

This aggregation-dependent fluorescence was subsequently exploited to construct a sequential ion-responsive system. The addition of Fe^3+^ to **ONA** selectively quenched its blue emission and generated the corresponding Fe-**ONA** metallogel, with a detection limit of 6.96 × 10^−8^ M. Metal coordination also increased the storage modulus, indicating reinforcement of the supramolecular network. Subsequent addition of H_2_PO_4_^−^ competitively bound Fe^3+^ and restored both the original supramolecular organization and fluorescence, affording a reversible ON–OFF–ON response that could be repeated over four cycles. This behavior enabled the construction of a fluorescence-based logic operation and an erasable fluorescent film, while the **ONA** xerogel additionally removed 92.26% of Fe^3+^ from aqueous solution. Overall, this study expanded the role of NI-based gels from passive fluorescent materials toward multifunctional platforms in which self-assembly, fluorescence and ion recognition are integrated within a single supramolecular system.

**Figure 7:**
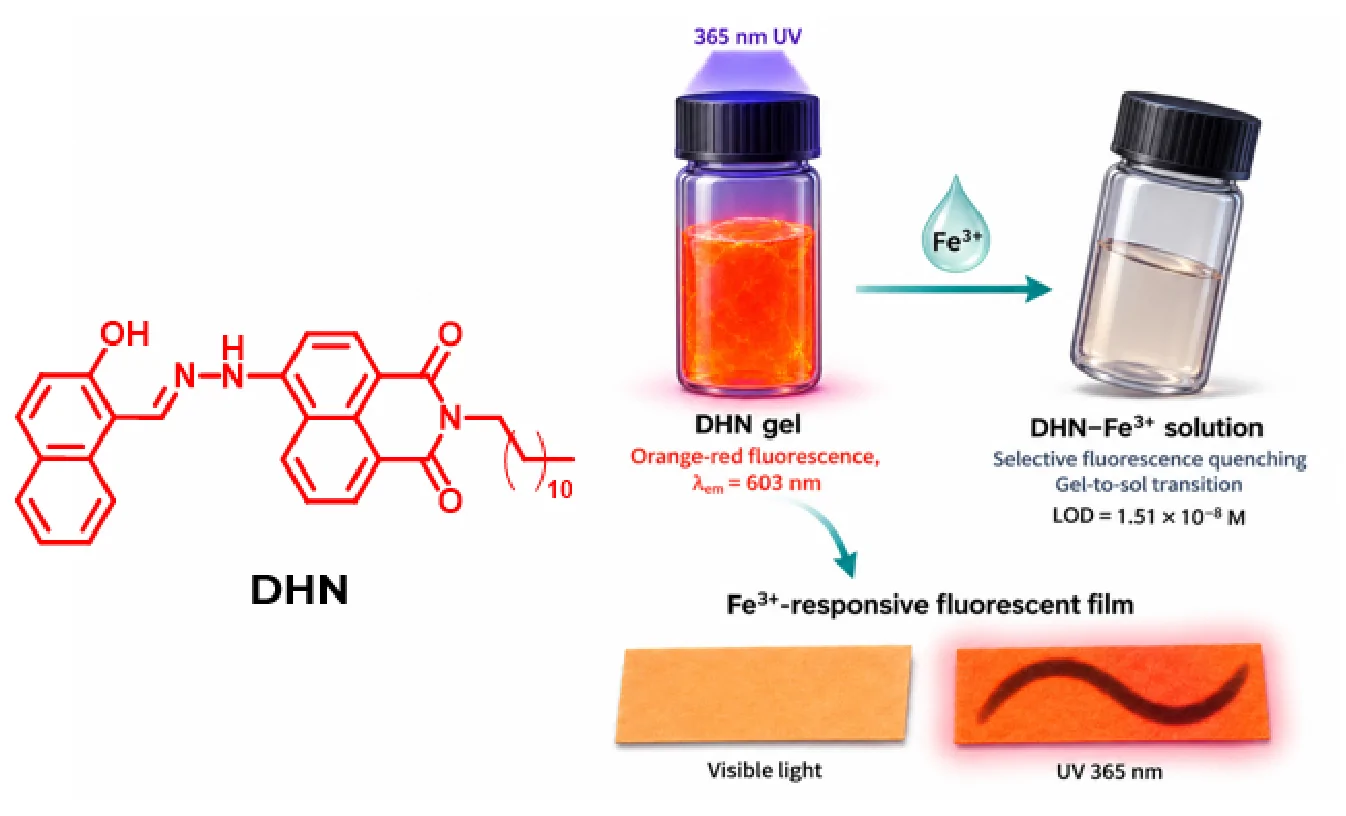
Original schematic representation of the Fe^3+^-responsive behaviour of the **DHN** supramolecular gel, showing selective fluorescence quenching, Fe^3+^-induced gel-to-sol transition and application as a fluorescent sensing film. Recreated from Ref. [42] with ChatGPT Sol 6.1.

Following the development of Fe^3+^-responsive NI gels, Liu et al. reported in 2023 the low-molecular-weight gelator **DHN**, in which an NI core was functionalized with a 2-hydroxynaphthalene unit through a Schiff-base linkage and combined with a dodecyl chain (Figure 7). **DHN** formed a stable supramolecular gel in DMF/H_2_O (10:3 *v*/*v*), displaying an intertwined fibrous morphology. In this system, the NI-containing aromatic framework played both structural and photophysical roles. XRD and single-crystal analysis supported π–π stacking between the aromatic naphthalene/naphthalimide units, with an intermolecular distance of approximately 3.45 Å, which cooperated with hydrogen bonding and van der Waals interactions to stabilize the supramolecular assembly. Interestingly, gelation was accompanied by an enhancement and bathochromic shift of the fluorescence from 575 nm in dilute solution to 603 nm in the gel, attributed to restricted molecular motion and aggregation of the conjugated chromophores [42].

As in the previously discussed 2022 system, the resulting gel was highly responsive to Fe^3+^ (Figure 7). Metal coordination disrupted the non-covalent interactions responsible for self-assembly, producing simultaneous fluorescence quenching and a gel–sol transition, with a detection limit of 1.51 × 10^−8^ M. This response was exploited to fabricate a fluorescent test strip for Fe^3+^-induced writing. Thus, although this work follows the increasingly recurrent strategy of Fe^3+^ sensing with NI-based gels, the combined structural and photophysical observations support a possible relationship between aromatic stacking of the NI-containing π-system, gel formation, and gelation-induced fluorescence enhancement.

**Figure 8:**
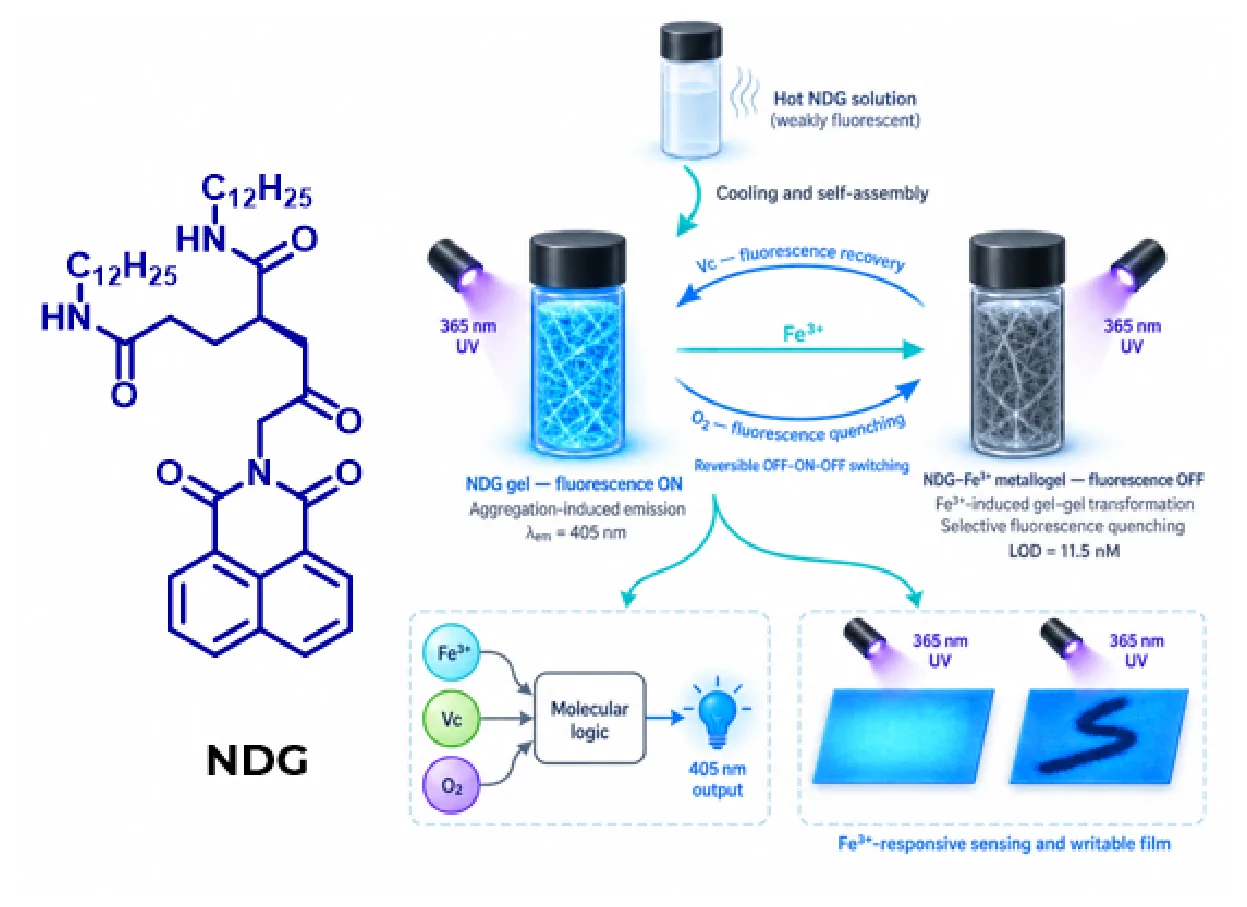
Original schematic representation of the self-assembly and functional response of the NI-based gelator **NDG**, showing aggregation-induced emission, Fe^3+^-induced gel–gel transformation and fluorescence quenching, reversible switching mediated by ascorbic acid (Vc) and O_2_, molecular-logic operation, and application as a fluorescent sensing and writable film. Recreated from Ref. [43] with ChatGPT Sol 6.1.

Following our overview, in 2024, Lian et al. further explored Fe^3+^-responsive NI-based gels through the design of **NDG**, an L-glutamine-containing NI gelator bearing amide functionalities and long C12 alkyl chains (Figure 8). The molecular components played complementary roles in self-assembly: the NI core acted as both the fluorescent unit and a π–π stacking motif, while the amide groups and alkyl chains promoted hydrogen bonding and van der Waals interactions, respectively. **NDG** formed thermoreversible organogels in several solvents and exhibited pronounced aggregation-induced emission enhancement, with intense blue emission at 405 nm emerging upon gelation. Interestingly, addition of Fe^3+^ selectively quenched this fluorescence without destroying the gel network, instead inducing a gel-to-gel transformation into the stable **NDG**–Fe^3+^ metallogel, with a detection limit (LOD) of 11.5 nM in the gel state. Structural analyses suggested that Fe^3+^ induced changes in the original π-stacked arrangement while retaining features consistent with lamellar organization, and XPS measurements suggested the involvement of the NI carbonyl oxygen atoms in metal coordination. The fluorescence response could also be reversibly switched through Fe^3+^/ascorbic acid/O_2_ cycles and translated into molecular logic and paper-based fluorescent sensing. Thus, compared with the Fe^3+^-induced gel–sol transition described in the preceding system, this work demonstrates how metal coordination can reorganize an NI-based supramolecular network without macroscopic gel disruption, providing a distinctive gel–gel sensing mechanism [43].

**Figure 9:**
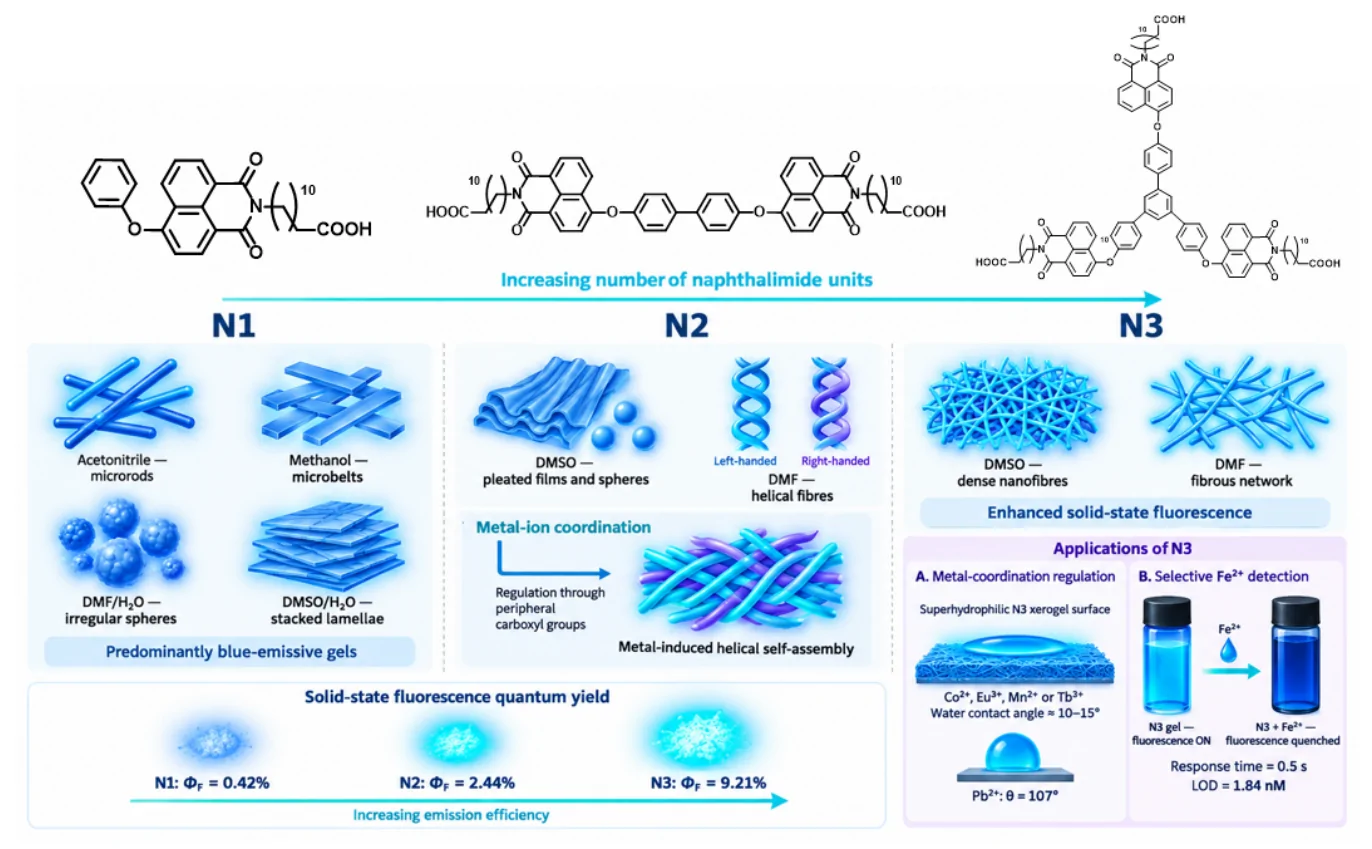
Original schematic comparison of the self-assembly behaviour of gelators **N1**–**N3** with increasing numbers of naphthalimide units, highlighting structure-dependent gel morphology and solid-state fluorescence, metal-coordination-mediated regulation of morphology and surface wettability, and the selective Fe^2+^ response of **N3**. Recreated from Ref. [44] with ChatGPT Sol 6.1.

Also in 2024, Wu et al. introduced a particularly systematic approach to elucidate the influence of the NI scaffold on supramolecular gel properties by comparing three gelators (**N1–N3**) containing one, two, and three NI units, respectively (Figure 9). Increasing the number of NI groups markedly affected gelation ability, morphology and fluorescence, producing diverse self-assembled structures ranging from rods and lamellae to fibrous and helical architectures. Spectroscopic and XRD analyses revealed solvent- and structure-dependent J- or H-type aggregation together with π–π stacking, highlighting the direct involvement of the NI π-system in controlling molecular packing. Interestingly, the solid-state fluorescence quantum yield increased from 0.42% for **N1** to 9.21% for **N3**, demonstrating that the number and spatial arrangement of NI chromophores can strongly modulate the emissive properties of the assembled material. Beyond fluorescence, metal coordination through the peripheral carboxyl groups provided an additional level of control over morphology and surface wettability, including the formation of metal-induced helical nanofibers and superhydrophilic surfaces. Among the three systems, **N3** also selectively detected Fe^2+^ through fluorescence quenching, with a response time of 0.5 s and a detection limit of 1.84 nM. Thus, rather than using NI exclusively as a fluorescent sensing unit, this work demonstrates how the number of NI chromophores can itself become a molecular design parameter for tuning self-assembly, photophysical behavior and macroscopic surface properties [44].

**Figure 10:**
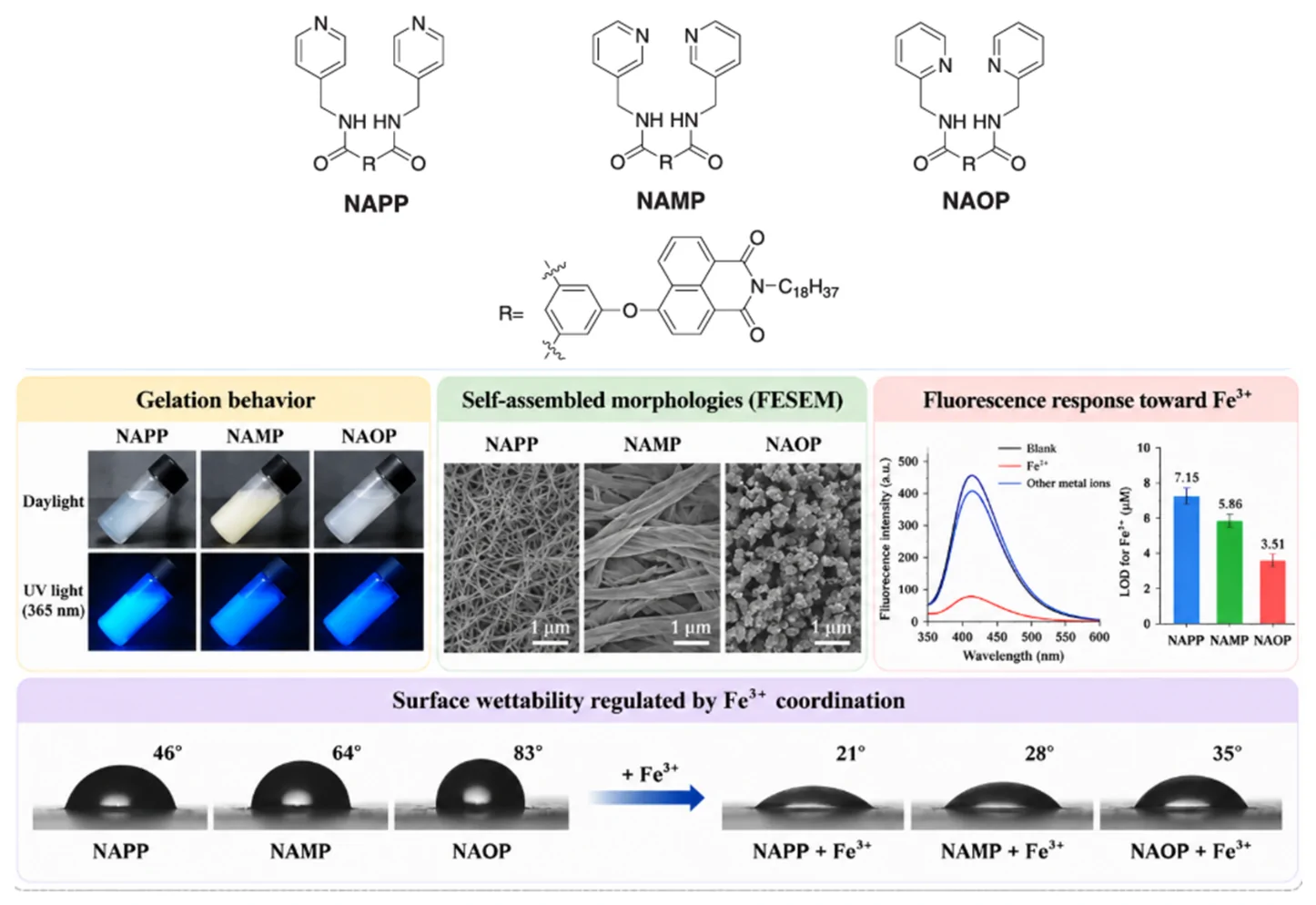
Molecular structures of the three 1,8-naphthalimide-based gelators (**NAPP**, **NAMP**, and **NAOP**) bearing different terminal pyridine isomers, together with their gelation behavior, fluorescence properties, and self-assembled morphologies. The selective fluorescence response toward Fe^3+^ and the modulation of surface wettability induced by metal-ion coordination are also illustrated. Recreated from Ref. [45] with ChatGPT Sol 6.1.

Finally also in 2024, Wu et al. investigated the influence of positional isomerism and metal-ion coordination on the self-assembly and functional properties of three 1,8-naphthalimide-based organogelators, **NAPP**, **NAMP**, and **NAOP**, bearing terminal 4-, 3-, and 2-pyridyl groups, respectively (Figure 10). All three derivatives exhibited gelation ability in different organic solvents and aqueous solvent mixtures, although their critical gelation concentrations (CGCs) and self-assembled morphologies were strongly dependent on the position of the pyridine nitrogen and the solvent composition. For instance, in methanol, **NAPP** and **NAOP** displayed CGC values of 8.33 mg mL^−1^, whereas **NAMP** required a higher concentration of 12.5 mg mL^−1^. Field-emission scanning electron microscopy (FESEM) revealed diverse supramolecular architectures, including entangled nanofibers, nanobelts, and irregular aggregates. FTIR and X-ray diffraction analyses indicated that hydrogen bonding and π–π stacking interactions played important roles in the self-assembly process, with the different pyridine substitution patterns influencing the molecular packing arrangements [45].

The resulting gels exhibited blue fluorescence under UV irradiation (365 nm), with emission maxima of approximately 425–430 nm in DMSO/H_2_O (10:1, *v*/*v*), compared with 437 nm in solution. Interestingly, **NAMP** and **NAOP** showed hypsochromic shifts in their absorption spectra upon gelation, consistent with the formation of H-type aggregates. Furthermore, the coordination ability of the terminal pyridine groups enabled the modulation of the supramolecular organization through the addition of metal ions. In particular, all three derivatives exhibited selective fluorescence quenching toward Fe^3+^ in DMSO solution, with LODs of 7.15, 5.86, and 3.51 μM for **NAPP**, **NAMP**, and **NAOP**, respectively. Metal-ion addition also induced substantial changes in the morphology and surface wettability of the corresponding xerogels (Figure 10). Overall, this study demonstrates how subtle structural modifications, such as the position of a pyridine nitrogen atom, combined with metal coordination, can be exploited to regulate gelation behavior, molecular packing, fluorescence response, and surface properties in NI-based supramolecular materials [45].

**Figure 11:**
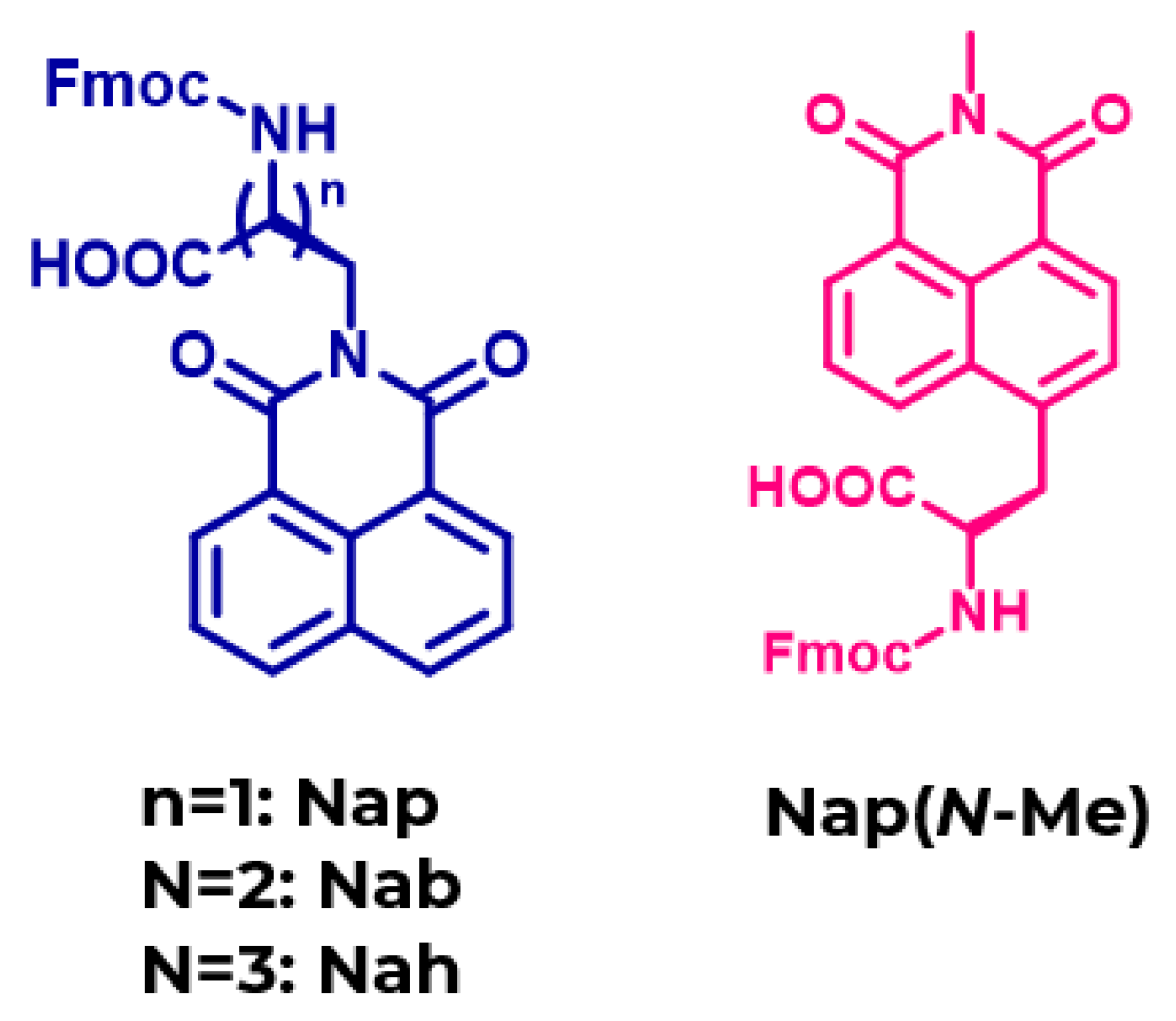
Chemical structures of 1,8-naphthalimide (NI) and the NI-derived amino acids **Nap**, **Nab**, **Nah**, and **Nap(N-Me)** investigated in Ref. [46].

A conceptually different strategy was introduced by Bertouille et al. in 2025 [46], who incorporated NI-derived unnatural amino acids directly into the self-assembling amphipathic peptide H-FQFQFK-NH_2_ to obtain fluorescent β-sheet-based hydrogels. Unlike previous peptide gelators in which NI was mainly introduced as a terminal or pendant fluorophore, this approach embedded the chromophore within the β-sheet assembly itself, allowing the peptide secondary structure to control the relative orientation of the NI units. The incorporation of NI simultaneously increased hydrophobicity and aromatic π–π interactions, improving gelation and mechanical properties; stable hydrogels were obtained at 1% *w*/*v* (<10 mM) directly in physiological saline without organic cosolvents. Remarkably, eight of the nine NI-containing hydrogels displayed aggregation-enhanced emission (AEE), demonstrating that β-sheet organization can orient the chromophores to minimize aggregation-caused quenching (ACQ) and favor emissive aggregation. This effect was particularly pronounced for SBL-HG-299, containing the **Nap(N-Me)** amino acid, whose fluorescence increased by more than 25-fold upon self-assembly (Figure 11). Importantly, NI incorporation also reinforced the material, with SBL-HG-299 reaching a storage modulus of approximately 696 Pa compared with ~46 Pa for the parent peptide hydrogel, while retaining thixotropic and injectable behavior. Thus, this work provides a particularly clear example of reciprocal cooperation between NI and the supramolecular matrix: the NI chromophore promotes peptide assembly and strengthens the hydrogel, whereas the β-sheet architecture controls NI packing and converts aggregation into enhanced fluorescence [46].

**Figure 12:**
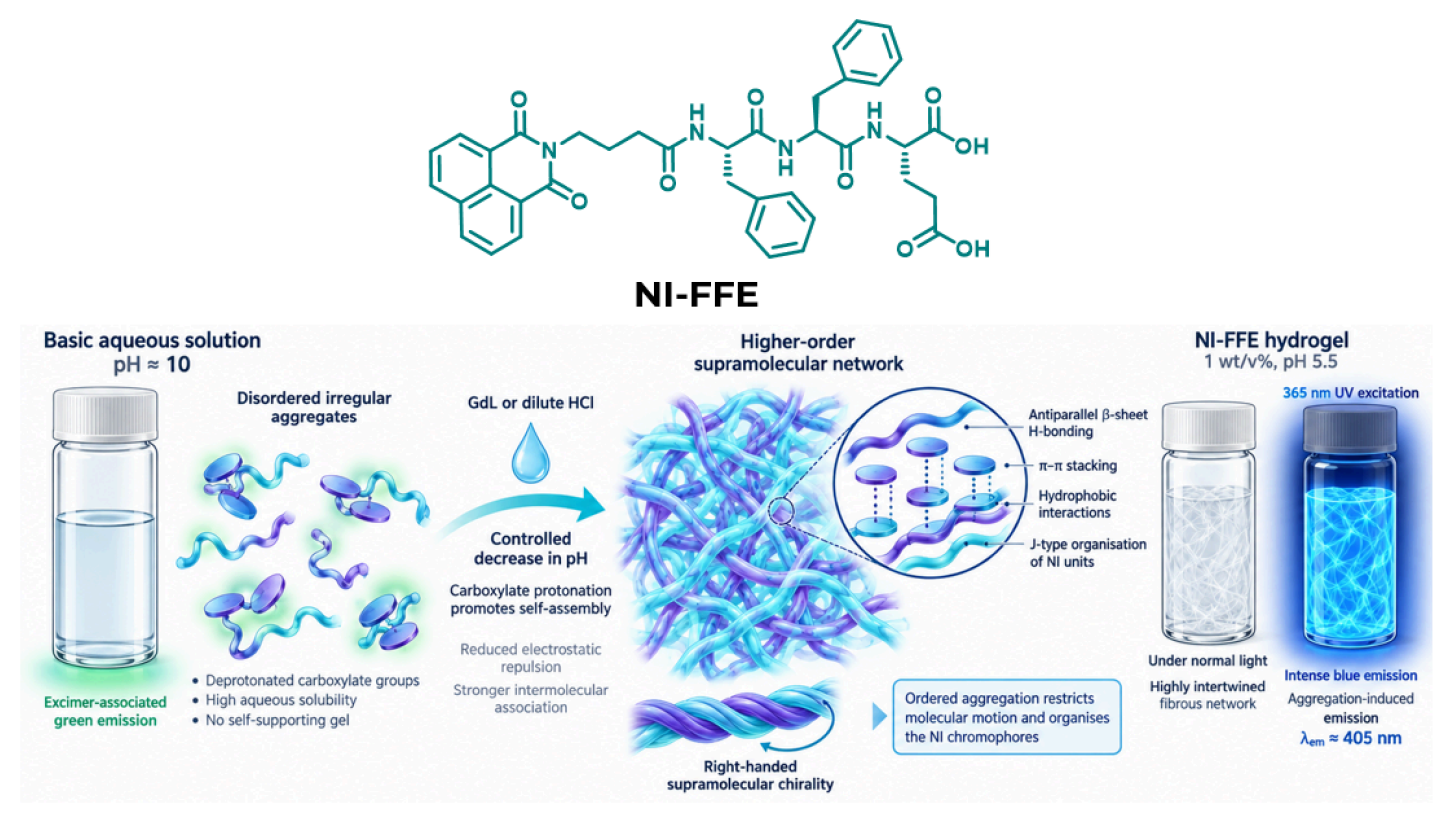
Original schematic representation of the pH-triggered self-assembly and fluorescence response of **NI-FFE**. Acidification of the basic aqueous precursor to pH 5.5 promotes the formation of an intertwined fibrous network and a self-supporting hydrogel at 1 wt/v%, accompanied by antiparallel β-sheet organisation, J-type aggregation of the NI units and intense blue emission under 365 nm irradiation. Recreated from Ref. [47] with ChatGPT Sol 6.1.

In a related peptide-based strategy, Kumari et al. developed in 2025 the ultrashort hydrogelator **NI-FFE**, in which NI was conjugated to the N-terminus of the tripeptide Phe–Phe–Glu (Figure 12). The molecular design assigned complementary functions to each component: the FF motif promoted β-sheet formation, glutamic acid provided aqueous solubility and pH responsiveness, whereas NI acted simultaneously as an AIE-active fluorophore and an aromatic driving force for self-assembly through hydrophobic and π–π interactions. At pH 5.5, **NI-FFE** formed a self-supporting hydrogel at 1 wt/v% displaying a highly intertwined fibrous network together with thixotropic and self-healing behavior. Spectroscopic measurements and molecular dynamics simulations supported antiparallel β-sheet formation and cooperative NI···NI and NI···phenylalanine aromatic interactions. Particularly remarkable was the photophysical response: self-assembly promoted J-type organization of the NI chromophores and intense blue AIE around 405 nm, with fluorescence enhancement exceeding 1000-fold relative to the molecularly dissolved state. The assembled NI units also displayed induced right-handed supramolecular chirality. Finally, the hydrogelator showed good biocompatibility across several cell lines, and its intrinsic NI fluorescence enabled direct visualization of cellular uptake. Together with the preceding peptide system, this study highlights the increasing potential of NI as a multifunctional component of peptide hydrogels, simultaneously promoting supramolecular organization and providing aggregation-responsive fluorescence for biomedical imaging [47].

**Figure 13:**
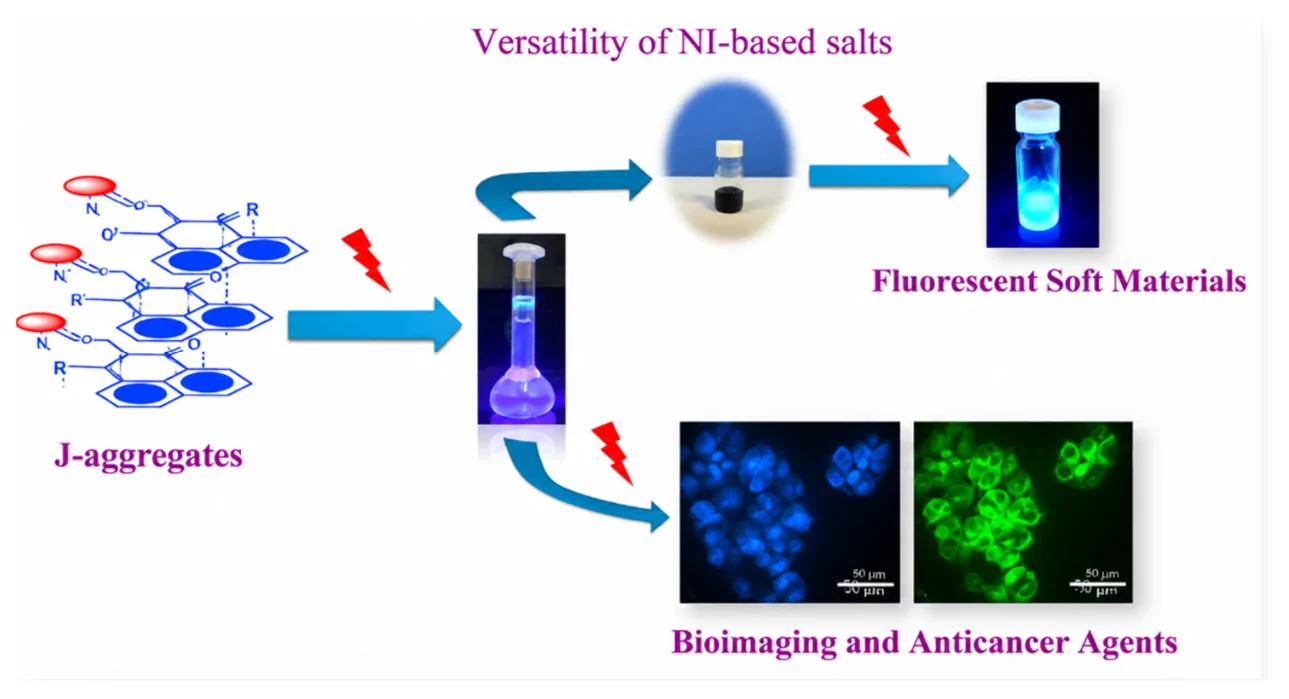
Schematic representation of the multifunctional behavior of NI-based ammonium salts, illustrating their self-assembly into J-aggregates, the formation of fluorescent supramolecular soft materials, and their potential applications as bioimaging and anticancer agents. Reproduced from Ref. [48] under the terms of the Creative Commons Attribution 4.0 International license (CC BY 4.0; https://creativecommons.org/licenses/by/4.0/, accessed on 16th September 2026). Copyright 2025 the authors; published by the American Chemical Society.

The recent evolution of NI-based therapeutic hydrogels was further illustrated by Billeci et al. in 2026 [48] through a family of fluorescent ammonium salts in which the alkyl chain length, spacer connecting the charged head to the NI core, and bromide/gluconate counterion were systematically varied. In close analogy with the earlier imidazolium-based systems, the NI unit retained its dual role as an aggregation-sensitive fluorophore and aromatic contributor to self-assembly, with the salts exhibiting aggregation-dependent spectroscopic changes attributed to J-type aggregation and AIE behavior in aqueous media (Figure 13). However, this study provided a more refined structure–property analysis, showing that relatively subtle changes in cation structure, hydrophobicity and counterion strongly influenced gelation, fluorescence, mechanical properties and biological activity. The salts acted as ambidextrous gelators in water and organic media, and several hydrogels displayed pronounced thixotropic and self-healing behavior. Most importantly, the gluconate derivatives showed enhanced cellular uptake, cytotoxicity and selectivity toward cancer cells, where they induced oxidative stress, mitochondrial dysfunction and predominantly apoptotic cell death. Release experiments further demonstrated that the hydrogel could liberate the NI-ammonium gelator at concentrations sufficient to exert its cytotoxic effect. Thus, this work closes the recent evolution of NI-based gels by integrating aggregation-dependent fluorescence, tunable supramolecular mechanics, bioimaging and therapeutic activity within a single self-assembled platform, reinforcing the potential of NI-based LMWGs as genuinely theranostic soft materials [48].

In order to facilitate a systematic comparison of the reported NI-based supramolecular gels, Tables S1 and S2 summarize the main structural features, gelation conditions, self-assembly mechanisms, photophysical and mechanical properties, stimuli-responsive behavior, and potential applications of the 12 studies discussed in this review. These tables aim to provide a clearer overview of the relationships between molecular design, supramolecular organization, and the resulting material properties.

Despite the significant advances reported in the selected examples in the characterization of NI-based supramolecular gels, certain limitations should be considered when interpreting their structural and photophysical properties. In particular, structural information obtained from single-crystal X-ray diffraction, xerogel characterization, or microscopy of dried samples does not necessarily reflect the molecular organization within the native gel state, as crystallization and solvent removal may induce structural rearrangements. Therefore, these techniques should be regarded as complementary approaches rather than direct evidence of the native gel network. Similarly, the assignment of specific aggregation modes, such as H- or J-aggregation, and photophysical phenomena, including AIE and AEE, requires appropriate spectroscopic evidence, since changes in colour or emission intensity alone are insufficient to establish these mechanisms unambiguously. In this context, the combination of complementary structural and spectroscopic techniques, particularly those performed under native gel conditions, will be essential for establishing more reliable relationships between molecular organization and functional performance.

## 3. Conclusions and Future Perspectives

The studies reported in this review between 2020 and 2026 demonstrate that NI constitutes a particularly versatile building block for the development of fluorescent supramolecular gels. Its combination of structural tunability, favorable photophysical properties, and extended aromatic character enables the NI unit to play a dual role within these materials, acting not only as an emissive chromophore but also as an active component of the molecular self-assembly process. Appropriate functionalization of the NI scaffold with hydrogen-bonding, coordinating, amino acid, peptide, or other recognition units provides access to a variety of LMWGs capable of generating organogels, metallogels, and, increasingly, water-compatible hydrogels.

The recent literature also highlights the intimate relationship between molecular structure, supramolecular organization, and photophysical behavior. Hydrogen bonding and π–π interactions represent the most recurrent driving forces for self-assembly, frequently complemented by van der Waals interactions, solvophobic effects, or metal coordination. Importantly, relatively subtle modifications of the molecular architecture, including the number of NI units or the introduction of specific peripheral functional groups, can substantially modify gelation ability, aggregate morphology, surface properties, and fluorescence. The organization of NI chromophores within the gel network can either promote or restrict non-radiative deactivation pathways, giving rise to aggregation-induced emission (AIE), aggregation-enhanced emission (AEE), gelation-induced fluorescence enhancement, or fluorescence quenching. Understanding and controlling these effects remains fundamental for the rational development of next-generation luminescent gels.

A major achievement during the period covered by this review has been the development of stimuli-responsive NI gels in which molecular recognition is translated into simultaneous optical and macroscopic responses. Metal-ion coordination, changes in pH, redox processes, and other chemical stimuli have been exploited to modulate fluorescence, supramolecular morphology, and gel stability, in some cases producing reversible gel–sol or gel–gel transitions. Such behavior has enabled applications ranging from metal-ion sensing to fluorescent displays, logic operations, and responsive surfaces. However, the recurrence of closely related metal-ion sensing strategies, particularly those involving iron ions, also indicates that further diversification of both recognition mechanisms and practical applications will be necessary to expand the impact of this family of materials.

One of the most significant recent developments is the transition from conventional LMW organogelators toward NI-containing hydrogels operating under increasingly biologically relevant conditions. The incorporation of amino acids and short peptide sequences has provided an effective strategy for combining the photophysical properties of NI with the biocompatibility and highly programmable self-assembly of peptide systems. Recent peptide-based hydrogels display aggregation-enhanced fluorescence together with improved mechanical properties, while thixotropic behavior and the possibility of recovering the gel network after mechanical disruption open opportunities for injectable materials. The emergence of systems combining fluorescence, bioimaging capability, and biological activity therefore represents an important expansion of the field toward biomedical applications.

Despite these advances, several limitations associated with NI-based gelators should be acknowledged. Their molecular design and synthesis may require multiple functionalization steps, increasing synthetic complexity compared with simpler low-molecular-weight gelators. Furthermore, gelation behavior is often highly sensitive to subtle structural modifications and experimental parameters, including solvent composition, concentration, and temperature, which can hinder reproducibility and rational optimization. From a photophysical perspective, unfavorable chromophore aggregation may lead to fluorescence quenching or other undesirable changes in emission properties, while the limited aqueous solubility of certain NI derivatives can restrict their applicability in biological environments. Although NI chromophores can exhibit favorable photostability, their long-term behavior under prolonged irradiation and practical operating conditions remains insufficiently investigated in many gel systems. Importantly, the specific contribution of the NI unit to gel formation should also be considered. Depending on the molecular architecture, NI may actively participate in supramolecular assembly through π–π interactions or primarily serve as a fluorescent and functional component within a gel-forming framework. Therefore, its incorporation does not necessarily improve gelation performance compared with simpler gelators, and the additional synthetic complexity should be justified by the functional advantages provided. Addressing these limitations, together with challenges related to scalability, long-term stability, and practical implementation, will be essential for advancing these materials toward real-world applications.

Beyond these general limitations, significant challenges remain regarding the detailed structural and photophysical characterization of NI-based supramolecular gels. In many reported systems, the relationship between molecular packing and excited-state behavior is still predominantly inferred from spectroscopic and morphological observations rather than established through detailed structural characterization. Future studies would benefit from combining microscopy and spectroscopy with diffraction, scattering, computational approaches, and time-resolved photophysical techniques to establish more quantitative structure–assembly–property relationships. In particular, time-resolved fluorescence spectroscopy could provide quantitative information on excited-state lifetimes and the relative contributions of different emissive species, whereas transient absorption spectroscopy could help identify short-lived excited states and competing relaxation pathways. Complementary structural investigations using small- and wide-angle X-ray scattering (SAXS/WAXS) and solid-state nuclear magnetic resonance (NMR) could provide further insights into nanoscale organization, molecular packing, and local intermolecular environments within the gel networks. Correlating these structural parameters with spectroscopic measurements under comparable experimental conditions would allow for a more rigorous assessment of the influence of supramolecular organization on fluorescence behavior. Parameters such as fluorescence quantum yields and excited-state lifetimes should also be systematically determined in the solution, aggregated, and gel states whenever possible, allowing for the effect of supramolecular organization on the excited-state dynamics of the naphthalimide chromophore to be properly understood.

From a photophysical perspective, another important limitation is the predominance of NI gels emitting in the blue-to-green spectral region. Molecular engineering strategies capable of extending π-conjugation and strengthening donor–acceptor interactions could facilitate the development of red- and near-infrared-emissive supramolecular gels. For example, extending the conjugated framework through the incorporation of additional aromatic or heteroaromatic units, or introducing stronger electron-donating substituents to enhance intramolecular charge-transfer (ICT) character, could contribute to reducing the optical energy gap and shifting the emission toward longer wavelengths. Furthermore, the incorporation of suitable excited-state intramolecular proton transfer (ESIPT) motifs may provide an alternative approach to achieving large Stokes shifts and potentially red-shifted fluorescence. Nevertheless, these structural modifications should be carefully balanced to preserve gelation ability and minimize aggregation-caused quenching and other non-radiative deactivation pathways. Such systems would be particularly attractive for biological imaging, where longer-wavelength excitation and emission offer important advantages, but could also broaden the technological scope of these materials beyond sensing and biomedical applications.

Regarding biomedical applications, further progress will require a more systematic assessment of the biological performance of NI-based supramolecular gels. Beyond preliminary cytotoxicity assays and fluorescence imaging experiments, future studies should evaluate the biocompatibility of both the gelators and their degradation products, including concentration-dependent toxicity, inflammatory responses, and long-term cellular effects. The degradation kinetics, biological clearance, and potential accumulation of gel-derived components should also be investigated using appropriate in vitro and in vivo models. In the case of injectable hydrogels, rheological characterization should be complemented by the evaluation of shear-thinning behavior, structural recovery after injection, injectability through needles of relevant dimensions, and mechanical stability under physiological conditions. These investigations would be essential to establish the safety and practical feasibility of NI-based gels for future biomedical applications.

Finally, the potential of NI-based gels as soft photonic materials remains largely unexplored. The hierarchical organization of fluorescent chromophores into elongated fibers and other anisotropic supramolecular structures provides an appealing platform for investigating directional energy transport, optical waveguiding, Förster resonance energy transfer, amplified spontaneous emission, and potentially other light-management phenomena. Combining these optical functionalities with the intrinsic responsiveness, processability, and dynamic character of supramolecular gels could ultimately lead to adaptive photonic materials whose optical properties can be reversibly controlled by external stimuli. Overall, the advances achieved during 2020–2026 demonstrate that NI-based supramolecular gels have evolved from relatively simple fluorescent organogelators toward increasingly sophisticated multifunctional soft materials. Continued integration of molecular design, supramolecular chemistry, quantitative photophysics, and application-oriented materials science should provide new opportunities for their development in sensing, biomedicine, and emerging photonic technologies.

## Data Availability

No new data were created or analyzed in this study. Data sharing is not applicable to this article.

## Supplementary Materials

The following supporting information can be downloaded at: https://www.mdpi.com/article/10.3390/gels12100933/s1. Table S1: Molecular design, gelation, assembly and application comparison. Table S2: Photophysical, rheological/thermal and stimulus-response comparison.
