# Supplementary material for: Recent Advances in 1,8-Naphthalimide-Based Supramolecular Gels (2020–2026)
Source: Gels. 2026 Oct 16;12(10):933. doi: 10.3390/gels12100933 (PMC13703986; doi:10.3390/gels12100933)
Supplement: Supplementary file 1 [file gels-12-00933-s001.zip › gels-4608079-supplementary.pdf]

# Recent Advances in 1,8-Naphthalimide-Based Supramolecular Gels (2020–2026)

**Table S1**. Molecular design, gelation, assembly and application comparison.

| Ref. | NI design | Gel medium/type | Minimum Gelation Concentration | Assembly/morphology | Application |
|---|---|---|---|---|---|
| **[37] Rizzo 2020** | NI–imidazolium salts; C10/C14, Br−/gluconate | Hydrogels; $H_2O$, aqueous buffers, $H_2O$/DMSO | [C14NIim][Br]: 2.0 wt% $H_2O$; 0.7 wt% Tris 1×; [C10NIim][Br]: 1.0 wt% Tris 1× | Networks (SEM); amphiphilic/ionic association | Bioimaging; cytotoxicity/theranostics |
| **[38] Misra 2020** | NI–dipeptide (NMI-GW-OMe) | Mixed water-miscible solvent/antisolvent gels | Ranging from 10 to 18 mM in aromatic solvents and long-chain aliphatic alcohols upon sonication. | J-type packing; peptide H-bonds; π interactions | Live-cell imaging |
| **[39] Lovitt 2021** | 4-Morpholino NI: L1 (picolyl), L2 (benzyl) | L1 $Mn^{2+}$/$Co^{2+}$ metallogels; L2 non-gelling | Not numerically established in main article text | Coordination-mediated assembly; pH-dependent aggregates | Responsive soft materials |
| **[40] Pei 2022** | Gallic-acid NI: disulfide GSSN vs GN | Organogels; DMSO, EtOAc | GSSN 20 mg/mL DMSO; 30 mg/mL EtOAc; GN 15 mg/mL DMSO/EtOAc | H-bonding, π–π stacking; distinct SEM morphologies | Reduction response; hydrophobic coatings |
| **[41] Jiang 2022** | NI fluorophore with pyridyl and amide functionalities (ONA) | ONA organogel; 1,2-propanediol | 1% w/v | Noncovalent assembly; xerogel networks | $Fe^{3+}$/$H_2PO_4^-$ sensing; $Fe^{3+}$ adsorption |
| **[42] Liu 2023** | DHN: hydroxynaphthalene-substituted NI | DMF/$H_2O$ gel | 1.5% w/v | H-bonding and aromatic stacking; crystalline reference | $Fe^{3+}$ sensing; strips |
| **[43] Lian 2024** | NDG: L-glutamine NI | Organogels; aromatic solvents, DMF, DMSO | Solvent-dependent; 1.5–7.5 mg/mL reported | H-bonds; π–π; van der Waals | $Fe^{3+}$ sensor; logic/display |
| **[44] Wu 2024 (S&I)** | N1/N2/N3 with 1/2/3 NI units | Solvent-dependent gels; metal coordination | N2: 25.0, 12.5, 7.14, 6.26 mg/mL (solvent-dependent); confirm mapping in source | N2 helical nanofibers with metal ions | $Fe^{2+}$ sensing; wettability |
| **[45] Wu 2024 (CSA)** | NAPP/NAMP/NAOP pyridine isomers | Methanol, acetonitrile, mixed solvents | MeOH: 8.33/12.5/8.33 mg/mL (NAPP/NAMP/NAOP); MeCN: 16.6 mg/mL (NAPP/NAMP) | Pyridine-position-controlled morphology; coordination | $Fe^{3+}$ sensing; surface switching |
| **[46] Bertouille 2025** | NI-amino-acid-containing β-sheet peptides | Aqueous physiological-saline hydrogels | 1% w/v gels characterized; lower threshold discussed for NI-modified peptide | β-sheet fibers; chromophore orientation | Potential imaging, sensing, printing |
| **[47] Kumari 2025** | NI–FFE tripeptide | Hydrogel; pH 5.5 | MGC 1% w/v | β-sheets; H-bonding; π–π; J-type packing | Cellular imaging; biocompatibility |
| **[48] Billeci 2026** | NI–ammonium salts; Br−/gluconate | $H_2O$, $H_2O$/DMSO, Tris, glycerol | [C14–NI-2]Br: 1.5 wt% $H_2O$, 1.0 wt% glycerol; [C14–NI-2]Glu: 7.0 wt% $H_2O$ | SEM networks; structure/counterion effects | Bioimaging; antitumoral activity |

**Table S2.** Photophysical, rheological/thermal and stimulus-response comparison.

| Ref. | Photophysical data | Mechanical / thermal data | Response and performance |
|---|---|---|---|
| **[37] Rizzo 2020** | Fluorescence in solution/gel; λem varies with medium; Φ/τ not numerically given in supplied main text | CGC table: [C14NIim][Br] Tgel 61 °C $H_2O$; 58 °C Tris 1×; rheology measured, G′/G″ values graphical | Solvent/ion structure |
| **[38] Misra 2020** | Solution peaks 386 nm in DMSO; broad excimer band 527 nm (solution context); AIE on aggregation | G′ on the order of $10^3$ Pa; G″ lower | Water fraction; AIE |
| **[39] Lovitt 2021** | L1/L2 solution emission ~508/511 nm (low polarity) to ~532/530 nm ($CH_3CN$); NOT gel λem | Metallogel 1: G′ = 1400 Pa, G″ = 200 Pa; Metallogel 2: G′ = 1520 Pa, G″ = 220 Pa (frequency sweeps; reported main-text values). | pH, heat, chemical stimuli |
| **[40] Pei 2022** | Absorption of GSSN gel ~350 nm (NOT emission); fluorescence aggregation quenching | G′ > G″ (tan δ < 1), no main-text numerical G′/G″ | GSSN reductant-responsive |
| **[41] Jiang 2022** | ONA gel λem 467 nm (λex 365 nm); blue AIE; Φ/τ not specified in main text | G′/G″ curves presented (Fig. 7); numerical moduli not tabulated | $Fe^{3+}$ turn-off, $H_2PO_4^-$ turn-on; $Fe^{3+}$ adsorption 92.26% |
| **[42] Liu 2023** | DHN: λem = 575 nm (dilute solution), 603 nm (gel); ΦF = 3.29% (gelation-enhanced state); τ = 1.4 ns (DHN gel before $Fe^{3+}$ addition). | No numerical G′/G″ found in main text | $Fe^{3+}$ LOD $1.51 \times 10^{-8}$ M gel; $7.58 \times 10^{-8}$ M aggregates |
| **[43] Lian 2024** | Blue AIE; λem/Φ/τ numerical values require figure/SI reading | No numerical G′/G″ established in main text | $Fe^{3+}$ LOD 11.5 nM gel; 30.8 nM diluted solution |
| **[44] Wu 2024 (S&I)** | DMSO solutions: ΦF(N1) = 2.14%, ΦF(N3) = 14.94% (N2 weakly emissive). Solids: ΦF(N1/N2/N3) = 0.42/2.44/9.21%. Solution τ(N1/N2/N3) = 1.85/1.82/1.04 ns. | No numerical G′/G″ established in main text | N3 $Fe^{2+}$ LOD 1.84 nM; response 0.5 s |
| **[45] Wu 2024 (CSA)** | Blue emission; solution and gel spectra; quantitative Φ/τ not established in main text | No numerical G′/G″ established in main text | $Fe^{3+}$ quenching; surface wettability switching |
| **[46] Bertouille 2025** | Nap/Nab solution λem ~427 nm (1 mM DMSO); 1% gel spectra separately reported | At 37 °C, 2-h time sweep: SBL-HG-063 G′ = 45.6 ± 1.2 Pa (phase angle 30.9 ± 1.5°); SBL-HG-299 G′ = 696.0 ± 119.3 Pa. For SBL-HG-299, phase angle decreased from 15.7 ± 0.8° to 5.8 ± 0.2° after injection/recovery. | Thixotropy; temperature and medium |
| **[47] Kumari 2025** | Blue luminescence; τ 2.82 ns monomer, 2.77 ns (50% $H_2O$ aggregates), 2.03 ns (90% $H_2O$ aggregates) | G′/G″ measured; numerical readings in plots, not tabulated in main text | pH-sensitive assembly; cell uptake |
| **[48] Billeci 2026** | Relative ΦF measured in water; λem/Φ in SI tables, not reproduced as numbers in main text | [C14–NI-2]Br: G′ = 2680 Pa in $H_2O$/DMSO 95:5 and up to 350 kPa in $H_2O$/DMSO 50:50 (distinct media); Tgel = 53 °C in $H_2O$ and 70 °C in glycerol. [C14–NI-2]Glu: Tgel = 36 °C in $H_2O$. | Mechanical shear; salt structure |
